# Estimated Intake of Potassium, Phosphorus and Zinc with the Daily Diet Negatively Correlates with ADP-Dependent Whole Blood Platelet Aggregation in Older Subjects

**DOI:** 10.3390/nu16030332

**Published:** 2024-01-23

**Authors:** Kamil Karolczak, Agnieszka Guligowska, Bartłomiej K. Sołtysik, Joanna Kostanek, Tomasz Kostka, Cezary Watala

**Affiliations:** 1 Department of Haemostatic Disorders, Medical University of Lodz, Ul. Mazowiecka 6/8, 92-215 Lodz, Poland; joanna.kostanek@stud.umed.lodz.pl (J.K.); cezary.watala@umed.lodz.pl (C.W.); 2 Department of Geriatrics, Healthy Aging Research Center (HARC), Medical University of Lodz, Pl. Hallera 1, 90-647 Lodz, Poland; agnieszka.guligowska@umed.lodz.pl (A.G.); bartlomiej.soltysik@umed.lodz.pl (B.K.S.); tomasz.kostka@umed.lodz.pl (T.K.)

**Keywords:** blood platelets, aggregation, cardiovascular risk, minerals, diet, ageing

## Abstract

The aggregation of blood platelets is the pivotal step that leads to thrombosis. The risk of thrombotic events increases with age. Available data suggest that minerals taken with diet can affect the course of thrombosis. However, little is known about the relationship between platelet aggregability and mineral intake with diet among elderly people. Thus, we evaluated the associations between the reactivities of platelets to arachidonic acid, collagen or ADP and the estimated quantities of minerals consumed as a part of the daily diet in 246 subjects aged 60–65 years (124 men and 122 women). The found simple (not-adjusted) Spearman’s rank negative correlations are as follows: 1. arachidonate-dependent aggregation and the amounts of potassium, zinc, magnesium, phosphorus, iron, copper and manganese; 2. collagen-dependent aggregation and the amounts of potassium, phosphorus, iron and zinc; and 3. ADP-dependent aggregation and the amounts of potassium, phosphorus and zinc. The negative associations between ADP-dependent platelet reactivity and the amount of potassium, phosphorus and zinc and between collagen-dependent aggregability and the amount of phosphorus were also noted after adjusting for a bunch of cardiovascular risk factors. Overall, in older subjects, the intake of minerals with diet is negatively related to blood platelet reactivity, especially in response to ADP. Diet fortification with some minerals may possibly reduce the thrombotic risk among elderly patients.

## 1. Introduction

Platelet aggregation is one of the stages of haemostasis (under physiological conditions) or thrombosis (under pathological conditions) closely related to platelet activation and adhesion to blood vessel wall, which together lead to the formation of a haemostatic plug. It is an evolutionarily developed process to inhibit blood loss after tissue damage. In atherogenic conditions, the key phenomenon involves the increased blood flow forcing into narrowed blood vessels [1] and damaging the endothelium followed by the exposure of proteins of the subendothelial layer. Proteins of this layer, such as collagen, have an activating effect on platelets and initiate adhesion followed by aggregation, which has a haemostatic effect under physiological conditions and a thrombogenic effect under pathological conditions [1,2]. Platelet aggregation includes interactions of platelets between themselves, as well as between platelets and a vascular wall. The main role in the platelet–platelet interactions is played by dimeric fibrinogen molecules that interact with platelet GPIIb/IIIa receptors, thus leading to platelet clumping, commonly referred to as platelet aggregation [2,3,4].

Platelet aggregation is a multifactorial process, and it depends on several agonists activated in a sequential manner. Collagen, as the main protein of the subendothelial layer exposed after endothelial denudation, seems to have the greatest thrombogenic potential among the proteins of the subendothelial layer [5,6]. Other platelet agonists, like arachidonic acid (AA) and ADP, are plasma-soluble substances secreted by blood platelets into the extraplatelet environment, where they may exert an autocrine-activating effect on platelets via G protein-coupled receptors (GPCRs) [7]. All the platelet agonists mentioned above—arachidonic acid, collagen and ADP—are commonly used in in vitro studies as the triggers of platelet aggregation [8,9], and this process can be possibly monitored by aggregometry [8,9,10]. This method enables the assessment of thrombotic risk both under experimental conditions [10] and in clinical screening [8,9].

The risk of thrombotic diseases is known to increase with age [11]. Little is known about age-related changes in platelet physiology, but it appears that a shift toward an increased susceptibility to activation and a decreased sensitivity to inhibitory factors is quite characteristic for platelets of older people [12], due to a higher prevalence of diabetes or lipid disorders in older men and women [13,14,15]. The optimisation of antiplatelet therapy in the elderly is one the biggest challenges of modern pharmacotherapy [16,17].

Thrombosis caused by platelet hyperreactivity is considered to be closely related to environmental factors, especially diet, and therefore, the changing of the nutritional habits is recommended as an important way to reduce cardiovascular risk in the elderly [18]. The term “cardiovascular risk” is understood differently in relation to cardioprevention with diet. In most cases, the markers of cardiovascular risk and the markers of the effectiveness of the protective effect of the diet are considered to be the indicators of carbohydrate–lipid metabolism, but blood platelets, as targets of such interventions, are not widely recognised [18,19,20]. Data on dietary ingredients and their potential impacts on platelets include selected groups of substances, such as *n*-3 polyunsaturated fatty acids, vitamins and polyphenols [21]. Numerous platelet studies have predominantly focused on polyphenols present in the diet [22]. Minerals are rarely included in this regard.

For minerals, it has already been shown that they have a disputable and controversial importance in the shaping of cardiovascular risk, and the ratio of benefits and dangers is not precisely known with respect to particular minerals [23,24,25,26]. Some reports suggest that mineral supplementation may be neutral in terms of cardiovascular prevention [27]. On the other hand, it is known that certain minerals positively modify cardiovascular risk factors. Potassium lowers blood pressure [28], and magnesium and calcium may have beneficial effects on the lipid profile [29]. In turn, the intake of phosphorus or sodium at higher concentrations is not recommended [30,31], while a higher magnesium intake is recommended [32]. The risk of heart attack is lowered with a higher copper intake [33]. It appears that many cardiovascular diseases can be reduced when adequate zinc levels are maintained [34].

Thus, it is clear that there are some discrepancies regarding the protective or detrimental effects of particular minerals. While some reports do not show the clear effects of minerals on cardiovascular health, others seem to clearly demonstrate negative or positive effects. In general, as in numerous other studies, references to platelets in the elderly are virtually non-existent.

For all minerals, we know that their supply and their level in the blood of older people are generally too low. This applies especially to calcium, magnesium [35], copper and zinc [36]. Among factors that potentially influence the mineral supply in older people, the non-biological ones can be indicated, like the area of residence (differences between urban and rural areas detected in South Korea for calcium, phosphorus and potassium) [37]. Additionally, the biological factors, such as somatosensory disorders, hormonal changes and the diseases of the digestive system, may significantly reduce appetite or food intake and, thus, may contribute to the failure to meet dietary mineral recommendations in older men and women [38,39]. The importance of deficits in the intakes of certain minerals by older people is indicated for some aspects of their health, such as an increased risk of frailty [40,41].

Thus, the issue of the relationship between mineral intake and platelet reactivity in older people seems important for the prevention of thrombotic diseases, but at the same time, it is underrepresented in the published studies. Therefore, in the present study, we attempted to assess the relationships between platelet reactivity and the mineral content in the typical daily diet of women and men aged 60–65.

## 2. Results

The characteristics of the final research group is summarised in Table 1 and Supplementary Table S1.

Based on the collected data (the recommended dietary allowances parameter (RDA) phosphorus, zinc, copper, iron, calcium and magnesium, it was calculated that 10, 47, 30, 51, 89 and 70% of the subjects had intakes of phosphorus, zinc, copper, iron, calcium and magnesium, respectively, below the recommended daily norm. Only 6% of the surveyed people had sodium intake below the recommended norm, and 87% of people had a potassium deficiency in their daily diet, what has benn caluclated using the parameter of adequate intake (AI). In the case of manganese content in the daily diet (estimated on the base of the daily diet questionnaire and presented in mg), we found that 5% of the study volunteers consumed meals containing manganese below the suggested daily allowance.

### Associations of the Amounts of Minerals in the Daily Diet and Platelet Reactivity to Arachidonate, Collagen and ADP in Older Subjects

First, in order to assess the potential statistical associations between the intake of dietary minerals (sodium, potassium, calcium, phosphorus, iron, zinc, copper, magnesium and manganese) with the daily diet and blood platelet reactivity, we calculated the simple (non-adjusted) Spearman’s rank correlation and, additionally, the bootstrap-boosted Pearson’s linear coefficients of correlations.

We observed in these simple associations that platelet reactivity to arachidonic acid, collagen or ADP regularly correlated in a negative and statistically significant manner with the amounts of potassium and zinc present in the daily diet. The correlations of platelet reactivity to arachidonic acid, collagen and ADP with daily intake of potassium and zinc were statistically significant in both methods of statistical analysis. The amounts of phosphorus correlated with arachidonic acid- and ADP-dependent aggregation of blood platelets, what has been shown with the both statistical methods of analysis. In case of collagen-induced platelet aggregation and amount of phosphorus in the typical daily diet the significant negative correlation was found only when Spearman’s rank correlation coefficient was used.

In the case of the dietary amount of iron, we noted negative and statistically significant associations with platelet reactivity to arachidonic acid and collagen, but no significant correlation with iron was evident for the ADP-dependent aggregation of blood platelets when Spearman’s rank correlation coefficients were calculated, but the bootstrap-boosted Pearson’s linear correlation coefficients revealed lack of significant associations with arachidonic acid or collagen-dependent platelet aggregation and significant negative association with ADP-induced platelet reactivity.

For all the examined minerals but sodium and calcium, we observed simple and significantly negative associations with arachidonate-induced platelet aggregation when we calculated the Spearman’s rank correlation coefficients.

The amounts of sodium, calcium, copper, magnesium and manganese exhibited no statistically significant associations with the collagen- and the ADP-induced aggregation of blood platelets (Table 2).

Next, we verified the statistical associations between the daily amounts of consumed dietary minerals and platelet reactivity after adjusting for the sets of selected confounding factors. We designed three sets of confounding variables and used them for the adjustment of simple associations. In the first group, we included morphological and biochemical serum parameters routinely diagnosed in order to assess general health status: sex, age, the count of white blood cells (WBC), the count of red blood cells (RBC), the concentration of haemoglobin (HGB), haematocrit (HCT), the count of blood platelets (PLT), mean platelet volume (MPV), plateletcrit (PCT), platelet distribution width (PDW), platelet–large cells ratio (P-LCR), the serum concentrations of triglycerides (TG), HDL cholesterol (HDL), LDL cholesterol (LDL), glucose (GLU) and uric acid (UA). The second group comprised the standard serum markers of cardiovascular risk, including variables describing platelet morphology, since the method of impedance aggregometry used in the present study highly depends on the platelet number. The set II consisted of the following variables: sex, age, PLT, MPV, PCT, PDW, P-LCR, TG, HDL, LDL, GLU and UA. However, in set III, we implemented sex, age and three dietetic variables: the intake of protein, the intake of fat and the intake of carbohydrates. All of these dietetic variables refer to the intakes with the typical daily diet.

In general, after adjusting the simple associations for the variables designed for sets I, II and III, we observed no significant associations between the reactivity of blood platelets to arachidonic acid and the daily dietary amounts of any tested dietary minerals. Thus, significant negative associations were shown by simple Spearman’s and Pearson’s coefficients for arachidonic acid-dependent platelet aggregation and the dietary amounts of potassium, phosphorus and zinc that disappeared in the course of the adjusting procedure.

In the case of the collagen-induced aggregation of blood platelets, we noted the significant negative associations with the daily dietary amount of phosphorus only after adjusting for the set II cardiovascular risk variables.

In the case of ADP-triggered platelet aggregation, we observed significant negative associations with the intake of potassium and phosphorus through the diet upon adjusting for the variables from the sets I and II of potentially confounding variables and for zinc upon adjusting for the variables of the set I (Table 3).

To further validate the above findings, we employed a canonical analysis that enabled us to estimate the contributions of the examined minerals to shape the variability of platelet aggregation dependent on either AA, collagen or ADP. First, the only overall significant canonical correlation coefficient was observed between the set of minerals and platelet aggregation dependent on ADP (R_C_ 0.27–0.29, *p* < 0.02) (Table S2). Second, rather consistently, zinc, potassium and phosphorus, with much less regular participation of iron, magnesium or calcium, were characterised by the highest values of the canonical analysis factor loadings, regardless of the set of adjusting variables used in the analysis (Supplementary Table S2). It means that, predominantly, these minerals significantly contributed to blood platelet responses to either AA, collagen or ADP. Moreover, in the case of ADP-induced platelet aggregation, such contributions appeared to play the biggest role, and in the present study, this type of platelet aggregation enabled the creation of the only significant model of canonical correlation with the sets of the studied minerals.

## 3. Discussion

The present study evaluated the relationship between the mineral content of the diet consumed during a typical 24 h period and the reactivity of platelets to arachidonic acid, collagen and ADP. The analysis was performed on a mixed-sex group of older subjects.

Simple correlations revealed few significant and negative correlations between the amount of mineral intake with the typical daily diet and platelet reactivity. In the case of arachidonate- and ADP-induced platelet aggregation, only potassium, phosphorus and zinc were found to exhibit a significant association, when both methods of simple (non-adjusted) statistical analysis of correlation were used. Similarly, in the case of collagen-triggered aggregation, potassium and zinc, but not phosphorus, appeared exhibited a significantly association when either simple (non-adjusted) Spearman’s rank correlation coefficients or bootstrap-boosted Pearson’s linear correlation coefficients were calculated. Some of these correlations remained significant after the adjustment for morphological and biochemical cardiovascular risk factors, but neither of them remained significant after the adjustment for the intakes of protein, carbohydrates and fat with the typical daily diet.

It has been suggested that blood platelets store potassium ions and release them in response to stimuli. About 20% of the potassium ions of platelet are encapsulated in alpha granules as a pool that is freely exchangeable and non-sequestered [42]. Potassium has been indicated to significantly reduce the risk of coronary thrombosis [43]. Three-day potassium supplementation with 60 mmol KCl/70 kg b.w./day in the form of tablets reduced platelet reactivity by an yet undeciphered pathway. It has been suggested that the sodium/potassium gradients across the platelet plasma membranes may be associated with platelet cytosolic calcium; indeed, platelet calcium homeostasis depends on membrane sodium/potassium gradients, and this association might be hypothetically responsible for the effects of potassium on platelet function [44]. The results presented by Lin and Young [43] and by Kimura and co-workers [44] seem to agree with our present outcomes. The reduction in platelet reactivity due to potassium supplementation or by the proper diet fortification with potassium (as we suggest in the present paper) may lead to a lower risk of coronary thrombosis [43,44].

Blood platelets express the intermediate conductance Ca^2+^-activated K^+^ channels, which, when activated, inhibit platelet aggregation by reducing platelet calcium signalling [45]. Many platelet-derived procoagulant processes, like secretion or integrin inside-out signalling, are modulated by Ca^2+^, and these may be influenced by potassium channels [46]. These processes may be responsible for the negative association observed herein between the daily potassium intake and the platelet readiness to respond to all of the tested platelet agonists (arachidonate, collagen and ADP) when simple correlations were calculated and only to ADP (but not to arachidonic acid or collagen) after the adjustment for both sets of cardiovascular risk factors, but not after the adjustment for the intakes of proteins, carbohydrates and fats.

A significant negative correlation between the potassium content estimated in meals representing a typical daily diet and platelet reactivity was noticed despite the fact that most of the examined subjects did not meet their potassium requirements (87% of volunteers had an adequate intake (AI) parameter below 100%).

Few studies have examined the role of dietary phosphorus on cardiovascular risk, identified as platelet reactivity. Undoubtedly, phosphorus is a pivotal factor in the regulation of platelet haemostasis, as it is found in important platelet-associated metabolites, such as ADP or polyphosphates [47]. Previous papers indicate a strong positive association between higher serum phosphorous levels and vessel calcification and, thus, higher cardiovascular risk, in patients with chronic kidney disease (CKD) [48]. In another study, it has been found that the preoperative serum levels of phosphorus allow the prediction of the risk of deep vein thrombosis in geriatric subjects [49]. Since none of our participants demonstrated CKD or deep venous thrombosis, it is difficult to compare our current results with those of the previous studies in this area. In our present study, we rather indicate that phosphorus can negatively affect platelet reactivity dependent on collagen or ADP and is neutral regarding platelet aggregability induced with arachidonic acid (when we look at the bootstrap-boosted partial correlation coefficents after adjusting for the cardiovascular risk factors). Negative correlations between the content of phosphorus in the typical daily diet and platelet reactivity to collagen or ADP are noticeable even after the adjustment for morphological and biochemical cardiovascular risk factors, but not after the adjustment for the intakes of proteins, carbohydrates and fats. When collagen is used as a platelet agonist, the statistical significance is maintained only after the adjustment for the set of cardiovascular variables excluding WBC and erythrocyte-associated variables, whereas in the case of platelet stimulation with ADP for both sets I and II of cardiovascular, morphological and biochemical variables, the correlations remain statistically significant.

Based on the analysis of the recommended dietary allowances parameter (RDA), it was found that for phosphorus, 90% of the tested subjects met or even exceeded the dietetic norm, so the high concentration of phosphorus in the diet and probably the efficiency of other mechanisms influencing its absorption in the body (which were not included in our studies) could have possibly caused a statistically significant relationship between the phosphorus content and platelet reactivity.

Taking into account our results and few papers published in the field [47,48,49], we are far from making robust suggestions regarding the platelet/cardiovascular safety of phosphorus dietary intake in older subjects. In the future experiments, these discrepancies should be clarified with special emphasis on cardiorenal background.

With the bootstrap-boosted partial correlation coefficients after adjusting for the cardiovascular risk factors it was found that the content of zinc present in the typical daily diet correlated negatively with the response of platelets to ADP only after the adjustment for the set I of cardiovascular risk factors. Thus, zinc certainly deserves further attention in the experiments conducted on larger groups of probands.

Based on literature reports, zinc would appear to have a significant effect on platelet activation and reactivity [50,51,52,53]. It can cross cell membranes from serum to platelet cytosol and thus contribute to the modulation of a number of signalling pathways that activate platelets [50], including the changes in PKC activity, thereby leading to full platelet aggregation [51]. Our results do not support this picture since platelet reactivity was found to be negatively related to the dietary zinc intake. It is possible that these differences may be due to the choice of the age group used in the study. However, our findings are in line with the data of another experiment, showing that a diet deficient in zinc significantly aggravates platelet aggregation [52] and that this may be due to an increase in oxidative stress in blood platelets deprived of zinc [53].

The requirement for zinc in the daily diet was met in 53% of the probands, as assessed by the analysis of the RDA index. In future studies, it is worth paying more attention to the importance of dietary zinc deficiency in shaping platelet reactivity.

There are very few results published to this date showing any impacts of other minerals that we studied in this investigation (including sodium, calcium, iron, copper, magnesium and manganese) on the overall reactivity of blood platelets.

High sodium intake is generally considered a cardiovascular risk factor. Indeed, a higher platelet aggregation has been noted among men and women with a higher sodium intake [54,55]. Our results do not support the view that sodium intake is associated with platelet aggregability. It is worth noting that our additional analysis of the AI parameter showed that only 6% of respondents had a sodium deficiency in the meals representing the typical daily diet, and as many as 94% met or exceeded the norm. Nevertheless, sodium did not appear to be significantly associated with platelet reactivity neither in the simple (non-adjusted) Spearman’s rank correlation nor the bootstrap-boosted Pearson’s linear coefficients of correlations or the bootstrap-boosted partial correlations coefficents.

Calcium plays a key role in the regulation of platelet haemostasis [56]. Dietary calcium exerts protective effects on blood platelets and decreases platelet aggregation, even when the diet is enriched with saturated fatty acids [57] or sodium [58]. Calcium supplementation reduces calcium mobilisation from intraplatelet calcium stores, thus attenuating the platelet response to agonists [59]. However, all these notions are not supported by the results presented in the current study, but there exists only a statistical tendency for the correlation between calcium intake and ADP-dependent aggregation, exclusively after the adjustment for the set I and II of cardiovascular variables (we refer here to the results of the bootstrap-boosted partial correlation coefficients).

In the case of calcium, the lack of a significant relationship between the consumption of this mineral with the typical daily diet and platelet reactivity may be due to the fact that most study participants consumed calcium-deficient meals—89% percent of respondents had an RDA for calcium below 100%.

Iron has been rarely associated with the function of mature platelets, and there is a striking scarcity of literature on any relationships between iron and blood platelet reactivity [60,61,62]. However, on the other hand, iron is known to play an important role in megakaryocyte differentiation in the bone marrow [60]. Little data are present on the relationship between exogenous iron and platelet activation. In premenopausal women with iron deficiency, iron supplementation reduces P-selectin expression on the surface of non-activated platelets and increases P-selectin expression on agonist-activated platelets, especially when stimulated by collagen-related peptide or ADP, but not U46619 [61]. Children suffering from iron deficiency-associated anaemia demonstrated a significantly lower platelet reactivity in response to collagen compared to non-deficient controls, and this reactivity increased after iron supplementation. Interestingly, an iron-stimulated increase in collagen-dependent aggregability was noted in both turbidimetric (optical) and whole blood (electrical) aggregometry [62]. Looking at the bootstrap-boosted partial correlation coefficients after adjusting for the cardiovascular risk factors we did not find any statistically significant associations of platelet reactivity and iron intake with the typical daily diet in older subjects.

In 51% of respondents, it was found that the RDA index calculated for the iron content in the typical daily diet was below 100%, which suggests a deficiency of this mineral. The degree to which iron deficiency may be important in shaping the relationship between the content of this mineral in the diet and the reactivity of platelets remains to be elucidated in further studies.

We are encouraged to hypothesise that copper may also have antiplatelet properties, since its deficiency has been reported to enhance platelet aggregability in rats [63]. Interestingly, copper was also found to significantly enhance the antiplatelet properties of acetylsalicylic acid [64,65]. Nevertheless, in the present study, contrary to the earlier published reports, no significant associations were observed between platelet reactivity and dietary copper in older subjects, with only one exception for the simple (non-adjusted) Spearman’s rank correlation coefficient found for arachidonic acid-induced aggregation of blood platelets.

The dietetic norm for copper in the daily diet was met by 70% of respondents (based on the analysis of the RDA parameter). It remains an open question to what extent the proper content of copper in the diet influences the reactivity of platelets.

Magnesium has been reported to have antiplatelet properties, including inhibiting platelet aggregation and adhesion via the formation of cAMP, reducing calcium ion influx and inhibiting the synthesis of proaggregatory eicosanoids, with different degrees of effectiveness with regard to platelet agonists [66,67,68]. However, in our study, the dietary amount of magnesium appeared to be not associated with platelet reactivity in older men and women in the analysis using the bootstrap-boosted partial correlation coefficients after adjusting for the cardiovascular risk factors. Only the simple (non-adjusted) Spearman’s rank correlation coefficient found for arachidonic acid-induced aggregation of blood platelets indicated on significant and negative association with intake of magnesium with the typical daily diet.

In the case of magnesium, for the majority of probands (70%), an RDA below 100% was identified based on the description of their typical meals, and this could have resulted in the lack of a significant relationship between the magnesium content in the diet and platelet reactivity.

Little data are available on the effects of manganese on blood platelets; however, it appears that it may enhance platelet activation [56]. Nevertheless, its ability to either induce or hamper platelet aggregations has not been noted so far, despite the presence of the manganese-induced formation of pseudopodia [69]. Our results obtained with the bootstrap-boosted partial correlation coefficients after adjusting for the cardiovascular risk factors rather suggests the lack of any association between the dietary amount of manganese and platelet reactivity.

Interestingly, as many as 95% of study participants fully met their demand for manganese or even exceeded it.

Thus, regarding the results of the current analysis, at least in the case of ADP-dependent platelet aggregability, phosphorus, potassium and zinc seem quite different from the other tested minerals, since they remain significantly and negatively associated with platelet reactivity in both the simple analysis of correlation and after the adjustment for both sets of cardiovascular risk factors. However, after adjustment for sex, age, the intake of protein, the intake of carbohydrates and the intake of fat with the daily diet these significant associations are no longer noticeable.

The leading contribution of potassium, phosphorus and zinc specifically to ADP-dependent platelet aggregability has been additionally confirmed by canonical analysis. Thus, three different statistical approaches indicate that higher intakes of potassium, phosphorus and zinc correlate negatively and significantly with ADP-induced platelet aggregation in older subjects.

All the potential discrepancies between our results presented above and the already published, very fragmentary outcomes may result from the essential demographic differences between the earlier studied populations and geriatric volunteers included in the present study. Moreover, a point of some significance is that our present study focuses on the dietary amounts of minerals in the typical daily diet and their relevance to blood platelet reactivity, while the earlier mentioned reports considered primarily the in vitro or, less frequently, the in vivo impacts of mineral concentrations in plasma on platelet functioning.

It should be noted that our findings are only statistical associations, and hence, they apparently do not fully reflect the cause–effect relationships between dietary minerals and platelet reactivity. However, it should also be emphasised that our study is one of the few (or even the very first) to assess the relationship between dietary minerals or mineral intake and blood platelet reactivity in the elderly. The older age group is rarely involved in platelet testing, despite the fact that elderly people are at a higher cardiovascular risk. We revealed in our present study that the correct fortification of the diet with some elements may be one of the ways to support the cardiovascular health of the elderly, but obviously, the effectiveness of this strategy seems to be clearly dependent on the biochemical pathway involved in platelet activation and on the presence of morphological and biochemical cardiovascular risk factors. In this regard, it seems that it is worth focusing our attention particularly on potassium, phosphorus and zinc as the most crucial minerals in shaping the ADP-dependent reactivity of blood platelets obtained from older subjects. However, due to a huge scarcity of papers presented in the literature up to date (only few papers dealing with this subject), and even more so, due to rather large discrepancies between the results presented hitherto in the available literature, and especially due to the indicated safety concerns regarding renal function, a clear indication of the supplementation with potassium, phosphorus and zinc as dietary ingredients inhibiting ADP-induced platelet aggregation in older subjects is not possible to be unrestrainedly announced, but the observed relationships seem to be provocative and challenging.

Our study has some limitations. We employed only one marker of platelet reactivity, i.e., platelet aggregation. We used dietetic data obtained from a short period of time, and thus, we are not able derive conclusions on the suspected longitudinal effects of dietary minerals on blood platelets. Moreover, blood samples were collected only once. The dietetic data gathered by us are the estimated values of minerals in the diet, but not the levels of minerals in the blood. On the other hand, it can be suspected that recording data for a short period of time enables obtaining more reliable dietetic data than through a questionnaire asking about a longer time interval. Dietetic data were recorded directly before blood sampling; thus, it is highly probable that blood platelets were recently under the influence of mineral intake (a period of time covered by the dietetic questionnaire) during sampling. Our study suggests that the estimation of minerals’ content in the diet may predict to some extent a higher or a lower reactivity of blood platelets. Nevertheless, these results should be verified on a larger group of probands.

## 4. Materials and Methods

### 4.1. Chemicals

Arachidonate, equine tendon collagen and ADP were purchased from Chrono-Log Corp. (Havertown, PA, USA). Physiological buffered saline (PBS) was procured from Avantor Performance Materials Poland S.A. (Gliwice, Poland). Blood sampling was performed using S-Monovette^®^ blood collection systems (Sarstedt, Nümbrecht, Germany).

### 4.2. Study Design

This study presents the results obtained in the subgroups of volunteers participating in the project “The occurrence of oxidative stress and selected risk factors for cardiovascular risk and functional efficiency of older people in the context of workload”. The project was funded by the Central Institute For Labour Protection-National Research Institute (Warsaw, Poland) and supervised by the Clinic of Geriatrics at the Medical University in Lodz (Poland). Recruitment was performed via announcements on local TV and radio and in newspapers. The two basic inclusion criteria comprised age within the range of 60 to 65 years, and a willingness to participate. The recruitment criteria are described in more detail elsewhere [70]. The target research group included roughly 300 subjects (equal sex proportions), aged from 60 to 65 years, and the final group included roughly 246 subjects (men—124; women—122. The detailed characteristics of the study group were presented in a previous publication [70]).

The experiments reported here were undertaken in accordance with the guidelines of the 1975 Helsinki Declaration for Human Research. The study was approved by the Committee on the Ethics of Research in Human Experimentation at the Medical University of Lodz. A written layout of the experiment with detailed information about its objectives, design, risk and benefits were presented to each of the volunteers during the course of recruitment. Informed written consent was obtained from each individual at the beginning of the experiment.

### 4.3. Blood Sampling, Isolation of Blood Plasma, Measurements of Blood Morphology and Serum Biochemistry

Blood was collected after overnight fasting and a 15 min rest period just before blood donation. Blood was collected by aspiration to vacuum tubes (Sarstedt, Nümbrecht, Germany) supplemented with 0.105 mol/L buffered sodium citrate (citrate/blood ratio = 1:9, *v*/*v*) for the measurements of platelet activation and reactivity. Other samples were collected in tubes coated with EDTAK_3_ for blood morphology analysis, or in tubes without any anticoagulant for isolating serum. In all cases, blood was collected from a peripheral vein cannulated with an 18-gauge needle.

Blood morphology was measured with a 5-Diff Sysmex XS-1000i haematological analyser (Sysmex, Kobe, Japan), while a DIRUI CS 400 analyser (Dirui, Changchun, China) was used to evaluate serum biochemical parameters. In order to obtain blood serum, the whole blood was collected in tubes without anticoagulant and was incubated for 30 min at 37 °C and centrifuged (2000× *g*/15 min./4 °C); the supernatant (serum) was aspirated and used in further analyses (basic blood serum biochemistry).

### 4.4. Whole Blood Impedance Aggregometry

Platelet aggregability was determined using a MultiPlate Analyzer (Dynabyte, Munich, Germany), as described previously [71]. In brief, the samples of citrated blood were left for 10 min at 37 °C to allow any reposition in order to avoid the interference of artefactual platelet activation caused by aspiration. Following this, 300 µL aliquots of whole blood were mixed with an equal volume of PBS, gently mixed and left at 37 °C for three minutes. Each sample was supplemented with the respective agonist, i.e., 0.5 mmol/L arachidonate, 1 µg/mL collagen or 10 µmol/L ADP, in order to trigger platelet aggregation. The recording of platelet clumping started immediately thereafter and was tracked for at least 15 min. Three measures of blood platelet reactivity were estimated: the area under the aggregation curve (AUC) and the maximal aggregation (A_max_) were measured directly with an aggregometer, and the third derivative measure of blood platelet clumping was calculated as (AUC×A_max_)/1000. This derivative measure reflects both the maximum platelet response to the action of an agonist and the stability of the formed platelet clump, and as such, it was employed in the evaluation of the associations.

### 4.5. Mineral Intake

The daily mineral intake was estimated by a qualified dietitian and nutritionist following a detailed analysis of the food intake reported by the participants. The data was collected using a 24 h recall questionnaire with a portion-sized photo album. The intake was recorded for three days, and the daily menu that was most representative of the typical diet was considered for further analysis.

To decrease the risk of errors from dietary recall, the participants were asked to prepare a list of food products, including snacks and beverages, that they ate on the day before the appointment. The interviewers were instructed not to judge the diet of the respondents. The method of interview was successfully used for the dietary evaluation among seniors [72].

None of the study participants used any kind of dietary supplements. The reported foods were analysed with Dieta 5.0 software (The National Food and Nutrition Institute, Warsaw, Poland) [73,74] to calculate energy intake and nutrient consumption. The stated amount of minerals (in mg) represents the level of consumption during a typical 24 h period (the menu was the most representative daily menu from diet reports recorded for three days).

### 4.6. Statistical Analysis

Continuous data were presented as either mean with SD or median with the interquartile range (from lower [25%] to upper [75%] quartile). Categorical data were presented as percentage fractions. Data normality was tested using the Shapiro–Wilk W-test, and the homogeneity of variance was verified with Levene’s test. In the case of variables for which data normality and variance homogeneity were not violated, the groups were compared with a non-paired Student’s *t*-test; otherwise, the Mann–Whitney *U*-test was used. The simple associations between variables were calculated with the Pearson’s or Spearman’s rank correlation coefficient. We used multiple regression analysis to estimate partial correlation coefficients upon the adjustment for the sets of potentially confounding variables. Canonical analysis was employed to associate two sets of variables: one, representing blood platelet function (aggregation in response to either AA, collagen or ADP) and the second, including the studied minerals. In this approach, canonical correlation coefficients reflected the significance of the relations between the two sets of variables, while the factor loadings represented the contributions of specific minerals in the shaping of the agonist-dependent platelet aggregation. In general, in order to avoid the risk of rejecting a null hypothesis by a pure chance, in all multivariate analyses, we employed bootstrap-boosted versions of statistical tests (10,000 iterations). Statistical analyses were performed with Statistica v.13 (Dell Inc., Tulsa, OH, USA), StatsDirect v.3.0.182 and R Package Software v. 4.4, using an algorithm for data resampling in multiple regression analysis written by one of the authors (J.K.).

## 5. Conclusions

In conclusion, our results suggest that the higher amounts of some minerals present in the daily diet of the elderly associate negatively with the reactivity of blood platelets in a mineral- and agonist-specific manner.

## Figures and Tables

**Table 1:** Blood morphology, serum biochemistry and chosen diet characteristics variables reported in the studied group.

Variable	Both Sexes (*n* = 246)	Males (*n* = 124)	Females (*n* = 122)	*p* Value (Less than)
Indices of blood morphology, biochemistry and blood platelet aggregation				
WBC (10^3^/mm^3^)	5.8 (5.0–6.9)	6.0 (5.0–6.9)	5.6 (5.1–6.8)	0.05 ^U^
RBC (10^6^/mm^3^)	4.5 ± 0.4	4.7 (4.4–4.9)	4.3 ± 0.3	0.0001 ^T^
HGB (g/dL)	13.8 (13.0–14.6)	14.4 (13.7–14.9)	13.3 ± 0.8	0.0001 ^U^
HCT (%)	39.8 (37.6–41.6)	41.1 (39.2–42.6)	38.5 ± 2.2	0.0001 ^U^
PLT (10^3^/mm^3^)	213.0 (181–243)	197.0 (168.5–228.5)	226.0 ±44.7	0.0001 ^U^
MPV (µm^3^)	11.3 (10.8–12.1)	11.2 ± 0.9	11.35 ± 1.0	n.s. ^T^
PCT (%)	0.2 (0.2–0.2)	0.22 (0.2–0.2)	0.26 ± 0.1	0.0001 ^U^
PDW (fl)	13.6 (12.4–15.6)	13.5 (12.1–15.2)	13.8 (12.7–16.3)	0.05 ^U^
P-LCR (%)	36.1 ± 7.7	35.7 ± 7.4	36.5 ± 8.4	0.05 ^T^
Lym (10^3^/mm^3^)	2.0 (1.6–2.4)	1.9 (1.5–2.2)	1.9 ± 0.5	n.s. ^U^
Mono (10^3^/mm^3^)	0.5 (0.5–0.7)	0.5 (0.5–0.7)	0.5 (0.4–0.6)	0.0001 ^U^
Neu (10^3^/mm^3^)	3.1 (2.6–3.9)	3.2 (2.5–3.8)	2.9 (2.5–4.0)	n.s. ^U^
Eo (10^3^/mm^3^)	0.2 (0.1–0.2)	0.1 (0.1–0.2)	0.1 (0.1–0.2)	0.05 ^U^
Baso (10^3^/mm^3^)	0.03 (0.02–0.03)	0.03 (0.02–0.03)	0.03 (0.02–0.03)	n.s. ^U^
Total cholesterol (mg/dL)	206.8 (173.8–237.3)	187.2 (168.7–218.3)	223.1 ± 49.9	0.0001 ^U^
Triglycerides (mg/dL)	111.2 (76.8–161.1)	111.2 (77.4–141.3)	110.5 (78.4–164.4)	n.s. ^U^
HDL cholesterol (mg/dL)	48.4 (41.0–59.3)	44.3 (40.2–51.0)	54.25 (44.1–63.4)	0.0001 ^U^
LDL cholesterol (mg/dL)	131.2 (103.4–156.5)	116.3 (101.4–139.0)	140.1 ± 39.6	0.001 ^U^
Glucose (mg/dL)	99.2 (91.4–108.3)	101.0 (93.8–111.6)	96.4 (89.0–105.4)	0.01 ^U^
Uric acid (mg/dL)	4.8 ± 1.2	5.4 (4.8–6.1)	4.3 (3.8–5.2)	0.0001 ^T^
AUC_AA	2486.5 (2075.5–2864.6)	2356.5 (1867.5–2643.5)	2652.0 (2217.0–2962.7)	0.001 ^U^
A_max__AA	128.0 (109.1–145.1)	123.7 (102.0–138.6)	134.0 (117.2–151.4)	0.01 ^U^
(AUC×A_max_)/1000_AA	319.2 (224.8–417.9)	291.9 (186.8–369.1)	354.5 (256.8–449.7)	0.001 ^U^
AUC_COL	2825.0 (2442.7–3326.2)	2680.0 (2273.7–3217.0)	2972.5 (2567.7–3463.2)	0.01 ^U^
A_max__COL	152.6 (133.5–177.6)	147.4 (123.8–167.9)	157.9 (137.2–184.9)	0.01 ^U^
(AUC×A_max_)/1000_COL	438.1 (328.6–587.7)	386.9 (287.9–535.5)	470.6 (356.8–636.8)	0.01 ^U^
AUC_ADP	2326.0 (1985.5–2746.4)	2167.0 (1808.7–2580.5)	2471.5 (2191.5–2812.5)	0.001 ^U^
A_max__ADP	122.3 (104.4–140.3)	113.4 (94.2–131.5)	128.1 (114.6–144.2)	0.001 ^U^
(AUC×A_max_)/1000_ADP	284.4 (206.4–376.9)	245.2 (170.3–343.5)	322.8 (254.4–408.3)	0.001 ^U^
Mineral intake				
Sodium (mg)	3338.9 (2435.6–4307.4)	3943.9 (2977.3–4790.6)	2795.8 (2092.6–3806.8)	0.0001 ^U^
Potassium (mg)	2975.5 (2275.4–4017.1)	3408.6 (2436.1–4342.4)	2805.0 (2239.0–3733.8)	0.05 ^U^
Calcium (mg)	589.6 (346.6–837.7)	610.5 (364.1–871.9)	573.0 (341.6–796.4)	n.s. ^U^
Phosphorus (mg)	1160.2 (921.3–1439.3)	1223.5 (963.6–1588.0)	1111.0 (873.9–1355.7)	0.01 ^U^
Magnesium (mg)	292.5 (231.2– 364.5)	309.0 (241.8–382.8)	269.8 (221.4–354.9)	0.05 ^U^
Iron (mg)	9.9 (7.9–13.4)	10.4 (8.2–14.1)	9.5 (7.4–12.1)	0.05 ^U^
Zinc (mg)	9.2 (7.2–11.8)	10.2 (8.2–13.2)	8.6 (7.0–10.7)	0.0001 ^U^
Copper (mg)	1.1 (0.8–1.5)	1.1 (0.9–1.5)	1.1 (0.8–1.4)	n.s. ^U^
Manganese (mg)	4.8 (3.6–6.9)	4.9 (3.7–7.3)	4.7 (3.5–6.1)	n.s. ^U^
Dietary indices				
Protein intake (g)	72.1 (54.9–85.8)	77.3 (59.8–94.0)	67.1 (50.0–81.4)	0.001 ^U^
Fat intake (g)	56.5 (39.8–83.0)	65.0 (46.2–95.4)	50.1 (34.3–66.9)	0.0001 ^U^
Carbohydrates intake (g)	224.2 (172.1–293.4)	252.5 (194.6–322.3)	199.4 (152.7–264.7)	0.0001 ^U^

Variables (not adjusted) presented as means ± SD or medians with interquartile ranges (from lower [25%] to upper [75%] quartile). Comparisons between men and women performed with the use of unpaired Student *t* test (^T^) or Mann–Whitney *U* test (^U^). The amounts of the consumed nutrients represent the levels of consumption with the daily diet (without supplements). Abbreviations used: AA, arachidonic acid; ADP, adenosine diphosphate; A_max_, the value of maximal platelet aggregation; AUC, area under aggregation curve; Baso, the number of basophils; COL, collagen; Eo, the number of eosinophils; HCT, haematocrit; HDL, high-density lipoproteins; HGB, the concentration of haemoglobin; LDL, low-density lipoproteins; LYM, the number of lymphocytes; Mono, the number of monocytes; MPV, mean platelet volume; n.s., *p* > 0.05, Neu, the number of neutrophils; PCT, plateletcrit; PDW, platelet distribution width; P-LCR, platelet–large cells ratio; PLT, platelet count; RBC, red blood cell count; and WBC, white blood cell count.

**Table 2:** Simple associations between the amounts of minerals present in the daily diet and the variables describing the aggregability of blood platelets in older subjects (men and women).

	AA	COL	ADP
Sodium (mg)	0.124 ^n.s.^/−0.096 ^n.s.^	−0.108 ^n.s.^/−0.027 ^n.s.^	−0.120 ^n.s.^/−0.116 *
Potassium (mg)	−0.175 ^##^/−0.147 ^#^	−0.130 ^#^/−0.143 ^#^	−0.159 ^#^/−0.109 ^##^
Calcium (mg)	−0.089 ^n.s.^/−0.080 ^n.s.^	−0.125 */−0.022 ^n.s.^	−0.085 ^n.s.^/−0.089 ^n.s.^
Phosphorus (mg)	−0.134 ^#^/−0.142 ^#^	−0.134 ^#^/−0.102 ^n.s.^	−0.130 ^#^/−0.179 ^##^
Iron (mg)	−0.155 ^#^/−0.102 ^n.s.^	−0.129 ^#^/−0.122 *	−0.108 ^n.s.^/−0.146 ^#^
Zinc (mg)	−0.175 ^##^/−0.168 ^##^	−0.160 ^#^/−0.142 ^#^	−0.187 ^##^/−0.215 ^##^
Copper (mg)	−0.149 ^#^/−0.122 *	−0.069 ^n.s.^/−0.061 ^n.s.^	−0.093 ^n.s.^/−0.102 ^n.s.^
Magnesium (mg)	−0.179 ^##^/−0.084 ^n.s.^	−0.08 ^n.s.^/−0.095 ^n.s.^	−0.085 ^n.s.^/−0.036 ^n.s.^
Manganese (mg)	−0.131 ^#^/−0.106 *	−0.074 ^n.s.^/−0.102 ^n.s.^	−0.063 ^n.s.^/−0.052 ^n.s.^

Results shown as simple (non-adjusted) Spearman’s rank correlation coefficients/the bootstrap-boosted Pearson’s linear correlation coefficients (estimated by the bootstrap resampling with replacement technique, 10,000 iterations). The reactivity of blood platelets was measured with impedance aggregometry (see ‘Materials and methods’) in response to arachidonic acid (AA), collagen (COL) or ADP (ADP) and expressed as (AUC×A_max_)/1000. The amounts of the consumed minerals (in mg) represent the consumption levels of minerals with the daily diet (without supplements) (for details see the section ‘Materials and methods’). The coefficients of correlations with a statistical significance of *p* < 0.05 or *p* < 0.01 are indicated with symbols ^#^ or ^##^, respectively. The statistical tendency (*p* = 0.05 or 0.05 < *p* < 0.1) is indicated with *; the statistically insignificant coefficients are marked as ‘^n.s.^’.

**Table 3:** Associations between the amounts of minerals present in the daily diet and the variables describing the aggregability of blood platelets in older subjects (men and women) after the adjustment for cardiovascular risk factors.

	AA	COL	ADP
Sodium (mg)	−0.034 ^n.s., I^ −0.055 ^n.s., II^ 0.068 ^n.s., III^	−0.050 ^n.s., I^ −0.057 ^n.s., II^ 0.072 ^n.s., III^	−0.050 ^n.s., I^ −0.053 ^n.s., II^ 0.086 ^n.s., III^
Potassium (mg)	−0.104 ^n.s., I^ −0.120 ^n.s., II^ −0.035 ^n.s., III^	−0.111 ^n.s., I^ −0.125 ^n.s., II^ −0.048 ^n.s., III^	−0.146 ^#, I^ −0.158 ^#, II^ −0.074 ^n.s., III^
Calcium (mg)	−0.086 ^n.s., I^ −0.093 ^n.s., II^ 0.009 ^n.s., III^	−0.097 ^n.s., I^ −0.107 ^n.s., II^ 0.004 ^n.s., III^	−0.117 *^, I^ −0.123 *^, II^ 0.006 ^n.s., III^
Phosphorus (mg)	−0.097 ^n.s., I^ −0.110 ^n.s., II^ 0.016 ^n.s., III^	−0.131 *^, I^ −0.144 ^#, II^ −0.040 ^n.s., III^	−0.136 ^#, I^ −0.146 ^#, II^ −0.015 ^n.s., III^
Iron (mg)	−0.046 ^n.s., I^ −0.059 ^n.s., II^ 0.014 ^n.s., III^	−0.097 ^n.s., I^ −0.110 *^, II^ −0.043 ^n.s., III^	−0.096 ^n.s., I^ −0.107 ^n.s., II^ −0.023 ^n.s., III^
Zinc (mg)	−0.017 ^n.s., I^ −0.116 *^, II^ −0.034 ^n.s., III^	−0.123 *^, I^ −0.132 *^, II^ −0.075 ^n.s., III^	−0.149 ^#, I^ −0.132 *^, II^ −0.080 ^n.s., III^
Copper (mg)	−0.097 ^n.s., I^ −0.106 ^n.s., II^ −0.036 ^n.s., III^	−0.061 ^n.s., I^ −0.071 ^n.s., II^ −0.003 ^n.s., III^	−0.092 ^n.s., I^ −0.098 ^n.s., II^ −0.010 ^n.s., III^
Magnesium (mg)	−0.057 ^n.s., I^ −0.074 ^n.s., II^ −0.042 ^n.s., III^	−0.117 *^, I^ −0.126 *^, II^ −0.073 ^n.s., III^	−0.013 ^n.s., I^ −0.026 ^n.s., II^ 0.042 ^n.s., III^
Manganese (mg)	−0.055 ^n.s., I^ −0.047 ^n.s., II^ −0.057 ^n.s., III^	−0.033 ^n.s., I^ −0.037 ^n.s., II^ −0.062 ^n.s., III^	0.016 ^n.s., I^ 0.019 ^n.s., II^ 0.009 ^n.s., III^

Results shown as the bootstrap-boosted partial correlation coefficients, estimated by the procedure of resampling with a replacement (10,000 iterations), after adjusting for the variables included in each of the following three sets of confounding variables: set I—sex, age, the count of white blood cells (WBC), the count of red blood cells (RBC), the concentration of haemoglobin (HGB), haematocrit (HCT), the count of blood platelets (PLT), mean platelet volume (MPV), plateletcrit (PCT), platelet distribution width (PDW), platelet–large cells ratio (P-LCR), the serum concentrations of triglycerides (TG), HDL cholesterol (HDL), LDL cholesterol (LDL), glucose (GLU) and uric acid (UA); set II—sex, age, PLT, MPV, PCT, PDW, P-LCR, TG, HDL, LDL, GLU and UA; set III—sex, age, the intake of protein, the intake of carbohydrates and the intake of fat with the daily diet. The reactivity of blood platelets was measured with impedance aggregometry (see ‘Materials and methods’) in response to arachidonic acid (AA), collagen (COL) or ADP (ADP) and recorded as the index (AUC×A_max_)/1000. The amounts of the consumed minerals (in mg) represent the consumption levels of minerals with the daily diet (without supplements) (for details see the section ‘Materials and methods’). The coefficients of correlations with a statistical significance of *p* < 0.05 are indicated with symbols ^#^. The statistical tendencies (*p* = 0.05 or 0.05 < *p* < 0.1) are indicated with *; the statistically insignificant coefficients are marked as ‘^n.s.^’.

## Data Availability

The data presented in this study are available on request from the corresponding author. The data are not publicly available due to ethical issues.

## Supplementary Materials

The following supporting information can be downloaded at: https://www.mdpi.com/article/10.3390/nu16030332/s1, Table S1. Medical history and medical treatment variables reported in the studied group; Table S2. Canonical correlations between the set of concentrations of diet minerals and blood platelet aggregation in older men and women.
