# Supplementary material for: Estimated Intake of Potassium, Phosphorus and Zinc with the Daily Diet Negatively Correlates with ADP-Dependent Whole Blood Platelet Aggregation in Older Subjects
Source: Nutrients. 2024 Jan 23;16(3):332. doi: 10.3390/nu16030332 (PMC10857292; doi:10.3390/nu16030332)
Supplement: Supplementary file 1 [file nutrients-16-00332-s001.zip › nutrients-2805162-supplementary.pdf]

Table S1. Medical history and medical treatment variables reported in the studied group.

Variable	Both sexes (*n* = 246)	Males (*n* = 124)	Females (*n* = 122)
Medical indices			
Age [years]	62.4 ± 1.7	62.9 ± 1.7	62.6 ± 1.6^{T*}
Current smoking [%]	23.4	23.4	21.3$^{F, n.s.}$
BMI [kg/m^2]	27.3 (24.7 – 30.3)	27.5 (25.06 – 30.4)	27.2 (24.4 – 30.04)$^{U, n.s.}$
WHR	0.92 (0.84 – 1.00)	0.99 (0.94 – 1.04)	0.84 (0.80 – 0.91)$^{U, ††}$
Hypertension [%]	46.1	53.2	39.3$^{F, *}$
Hypercholesterolaemia [%]	62.7	58.8	67.2$^{F, n.s.}$
Type 2 diabetes mellitus [%]	9.3	11.3	7.4$^{F, n.s.}$
Myocardial infarction in the past [%]	1.6	2.4	0.8$^{F, n.s.}$
Stroke in the past [%]	2.8	4.0	1.6$^{F, n.s.}$
Osteoporosis [%]	11.3	2.4	20.5$^{F, ††}$
Diseases of stomach and duodenum [%]	32.4	27.4	37.7$^{F, *}$
Cancer in the past [%]	6.8	5.6	8.2$^{F, n.s.}$
Ophtalmologic diseases [%]	20.2	20.1	20.5$^{F, n.s.}$
Depression [%]	14.9	9.7	20.5$^{F, \#}$
Chronic obstructive pulmonary disease [%]	12.1	10.5	13.9$^{F, n.s.}$
Joints diseases [%]	48.2	44.3	52.5$^{F, n.s.}$
Medicines taken currently			
Blocker of histamine receptor H2 [%]	0.0	0.0	0.0$^{F, n.s.}$
Acetylsalicylic acid [%]	0.0	0.0	0.0$^{F, n.s.}$
Clopidogrel or ticlopidine	0.0	0.0	0.0$^{F, n.s.}$
Acenonocoumarol [%]	0.0	0.0	0.0$^{F, n.s.}$
Nitrates [%]	0.4	0.8	0.0$^{F, n.s.}$
Beta blockers [%]	19.5	19.3	19.7$^{F, n.s.}$
Digoxin [%]	0.0	0.8	0.0$^{F, n.s.}$
Angiotensin converting enzyme inhibitors [%]	18.2	18.5	18.8$^{F, n.s.}$
Calcium channel blockers [%]	10.6	13.7	7.4$^{F, *}$
Indapamide [%]	10.5	12.1	9.0$^{F, n.s.}$
Spironolactone [%]	0.8	0.8	0.8$^{F, n.s.}$

Sartans [%]	5.7	4.0	6.5$^{F, n.s.}$
Thiazide [%]	3.2	1.6	4.1$^{F, n.s.}$
Amiloride [%]	0.4	0.0	0.8$^{F, n.s.}$
Torsemide [%]	0.4	0.8	0.8$^{F, n.s.}$
Eplerenone [%]	0.0	0.8	0.0$^{F, n.s.}$
Alpha blockers [%]	5.7	11.3	0.0$^{F, ††}$
Statins [%]	17.0	19.3	14.7$^{F, n.s}$.
Fibrates [%]	2.4	0.8	4.0$^{F, *}$
Bisphosphonates [%]	2.4	0.0	4.9$^{F, \#}$
Allopurinol [%]	2.4	4.0	1.6$^{F, n.s.}$
Insulin [%]	2.4	4.8	0.0$^{F, \#}$
Metformin [%]	6.5	6.5	6.6$^{F, n.s.}$
Gliclazide/glimepiride [%]	3.7	5.6	1.6$^{F, *}$
Steroids [%]	1.6	0.0	3.3$^{F, *}$
Methotrexate [%]	1.2	0.8	1.6$^{F, n.s.}$
Non-steroidal anti-inflammatory drugs [%]	2.0	2.4	1.6$^{F, n.s.}$
Beta mimetics [%]	3.2	2.4	4.1$^{F, n.s.}$
Antihistamines [%]	1.6	0.0	3.2$^{F, *}$
Antidepressants [%]	3.2	1.6	4.9$^{F, n.s.}$
Neuroleptics [%]	0.8	0.8	0.8$^{F, n.s.}$
Vinpocetine/nootropics [%]	3.7	4.0	3.3$^{F, n.s.}$
Trimetazidine [%]	0.8	0.8	0.8$^{F, n.s.}$
Mesalazine [%]	0.4	0.0	0.8$^{F, n.s.}$
Trimebutine [%]	0.8	0.8	0.8$^{F, n.s.}$
Diosmin [%]	2.4	0.0	4.9$^{F, \#}$
Levodopa [%]	0.4	0.8	0.0$^{F, n.s.}$

Continuous variables presented as means ± SD, medians with interquartile ranges (from lower [25%] to upper [75%] quartile). Categorical variables (not adjusted) presented as percentage fractions of a whole group of investigated patients.

Comparisons between men and women performed with the use of unpaired Student *t* test (^{T}), Mann–Whitney *U* test (^{U}) or (^{F}) Fisher's exact test. $^{*}P \le 0.05$, $^{\#}P < 0.01$, $^{††}P < 0.0001$, $^{n.s.}P > 0.05$..

Abbreviations used: BMI. body mass index; WHR, waist-hip ratio.

Table S2. Canonical correlations between the set of concentrations of diet minerals and blood platelet aggregation in older men and women

platelet aggregation (dependent variable)	*total redundance [%]*	*explanatory variables (set 2)*	*extracted variance [%]*	*total redundance [%]*	*canonical correlation*	*canonical determination [R2]*	*P*	*Wilks' lambda*	*best contributors expl var*
**set I**									
AA-dependent	6.859%	*microelements*	26.179%	1.844%	0.257	0.066	0.150	0.931	Zn, K, P
collagen--dependent	8.779%	*microelements*	22.351%	1.952%	0.290	0.084	0.072	0.912	Zn, P, K, Mg, Fe
ADP-dependent	10.662%	*microelements*	20.690%	2.226%	0.323	0.104	0.020	0.893	Zn, K, P, Fe
**set II**									
AA-dependent	6.895%	*microelements*	26.132%	1.842%	0.257	0.066	0.149	0.931	Zn, K, P
collagen--dependent	8.836%	*microelements*	22.180%	1.944%	0.291	0.085	0.069	0.912	Zn, P, K, Mg, Fe
ADP-dependent	10.712%	*microelements*	20.615%	2.223%	0.324	0.105	0.018	0.893	Zn, K, P, Fe
**set III**									
AA-dependent	6.857%	*microelements*	26.091%	1.825%	0.257	0.066	0.147	0.931	Zn, K, P
collagen--dependent	8.837%	*microelements*	22.164%	1.946%	0.291	0.084	0.074	0.912	Zn, P, K, Mg, Fe
ADP-dependent	10.717%	*microelements*	20.653%	2.229%	0.323	0.104	0.021	0.893	Zn, K, P, Fe

R^2 is a squared canonical correlation coefficient (canonical determination), which is relevant to overall variance between canonical variables. Total redundancy is relevant to the variance between the canonical variable for set 2 (minerals of the study) and the variable describing whole blood platelet response to the agonization with AA, collagen or ADP; it shows the representation of a given canonical variable by the explanatory variables of the set 2. Extracted variance is a variance between a given canonical variable and the variables that build it up; it reflects how well a given canonical variable represents a given set of variables. The Wilks' lambda is the parameter reflecting the contribution of a set 2 to the explaining of the variance of a set 1; the lower is the Wilks' lambda,

the higher is the contribution. Canonical correlation was calculated with the use of the bootstrap-boosted procedure of resampling with replacement (10000 iterations). The following microelements were included in the set 2 (microelements): sodium (Na), potassium (K), calcium (Ca), phosphorus (P), magnesium (Mg), iron (Fe), zinc (Zn), copper (Cu) and manganese (Mn).
